# Competition Enhances the Effectiveness and Motivation of Attention Rehabilitation After Stroke. A Randomized Controlled Trial

**DOI:** 10.3389/fnhum.2020.575403

**Published:** 2020-09-30

**Authors:** María Dolores Navarro, Roberto Llorens, Adrián Borrego, Mariano Alcañiz, Enrique Noé, Joan Ferri

**Affiliations:** ^1^NEURORHB, Servicio de Neurorrehabilitación de Hospitales Vithas, Fundación Vithas, València, Spain; ^2^Neurorehabilitation and Brain Research Group, Instituto de Investigación e Innovación en Bioingeniería, Universitat Politècnica de València, València, Spain

**Keywords:** attention, competition, motivation, competitiveness, stroke, cognitive rehabilitation, group-based interventions, virtual reality

## Abstract

Attention deficits are among the most common cognitive impairments observed after experiencing stroke. However, a very limited number of studies have investigated the effectiveness of interventions that specifically focus on the rehabilitation of attention deficits among subjects with impaired attention. Although several interventions have included the use of computerized programs to provide dynamic stimuli, real-time performance feedback, and motivating tasks, existing studies have not exploited the potential benefits of multi-user interactions. Group-based and competitive interventions have been reported to be more enjoyable and motivating, depending on individual traits, and may potentially be more demanding, which may increase their effectiveness. This study investigated the effectiveness and motivating abilities of an intervention specifically designed to address attention deficits. This intervention combined paper-and-pencil tasks and interactive, computerized, multi-touch exercises, which were administered, either non-competitively or competitively, to a group of 43 individuals with chronic stroke. The mediating effects of competitiveness were evaluated for both intervention effectiveness and motivation. Participants were randomly sorted into two groups and underwent 20 one-hour group-based sessions, during which they either worked individually or competed with peers, according to their group allocation. Participants were assessed before and after the intervention, using the Conners' Continuous Performance Test, the d2 Test of Attention, the Color Trail Test, the Digit Span Test, and the Spatial Span Test. The competitiveness and subjective experiences of the participants after the intervention were investigated with the Revised Competitiveness Index and the Intrinsic Motivation Inventory, respectively. The results showed that participants who competed demonstrated significantly greater improvements in all cognitive abilities, except for divided attention, and reported greater enjoyment than their non-competitive peers. Both groups reported comparable levels of perceived competence, pressure, and usefulness. Interestingly, the competitiveness of the participants did not alter either the effectiveness or the subjective experience of the intervention. These findings suggest that competition might enhance the effectiveness and enjoyment of rehabilitation interventions designed to address attention deficits in individuals post-stroke, regardless of their level of competitiveness and without having a negative effect on their perceived pressure and competence.

## Introduction

The distributed neural networks that sustain attention are particularly vulnerable to brain injuries caused by stroke (Raz and Buhle, 2006; Petersen and Posner, 2012; Rinne et al., 2013). As a consequence, impaired attention is one of the most common post-stroke deficits, with a prevalence ranging from 46 to 92%, during the acute phase (Stapleton et al., 2001), which persists years after injury in 20 to 50% of cases (Hyndman and Ashburn, 2003; Barker-Collo et al., 2009). Attention deficits can impact higher cognitive functions, such as working memory, executive function, and language (Lezak et al., 2004; Cumming et al., 2012), in addition to effects on daily functioning. Deficits in selective and divided attention have been shown to affect both the motor and social aspects of daily functioning (Hyndman and Ashburn, 2003), and sustained attention at 2 months post-stroke has been shown to predict functional recovery 2 years after the onset (Robertson et al., 1997).

Despite the functional repercussions associated with attention disorders after stroke, a limited number of studies have focused on the rehabilitation of attention. Instead of specifically addressing this skill, most interventions focus on general cognitive function, basing on the assumption that improvements in general function will have positive effects on attention (Cicerone, 2005; Cicerone et al., 2011). A recent update of a review examining the effectiveness of interventions designed to address attention deficits failed to identify any new randomized controlled studies that have been performed during the last 6-year period (Loetscher et al., 2019). Thus far, only six studies have been identified that aimed to restore or compensate for attentional impairments of individuals with demonstrable or self-reported attentional deficits post-stroke (Sturm and Willmes, 1991; Schöttke, 1997; Röhring et al., 2004; Westerberg et al., 2007; Barker-Collo et al., 2009; Winkens et al., 2009). The results showed that participation in a rehabilitation program resulted in beneficial effects on measures of divided attention, assessed immediately after treatment (with a low certainty of evidence), but not when assessed 3 to 6 months after the intervention (Loetscher et al., 2019). Treatment did not result in any other convincing effects for other measures of attentional skills, compared with usual care. Most interventions have used computer-based tasks, either alone or in combination with paper-and-pencil tasks, which were administered either at the clinic (Sturm and Willmes, 1991; Schöttke, 1997; Barker-Collo et al., 2009) or at home (Röhring et al., 2004; Westerberg et al., 2007; Winkens et al., 2009).

The advantages of computerized training over paper-and-pencil tasks include the provision of dynamic stimuli, which might be especially relevant for challenging attentional skills (Svaerke et al., 2019), and real-time performance feedback (Kueider et al., 2012). In addition, the exercise contents can be more easily adjusted to fit the clinical conditions of each participant, while providing engaging and motivating objectives through gamification (Kueider et al., 2012). Gamified objectives can be particularly interesting for individuals post-stroke, as clinical apathy has been linked to a lack of response to motivational stimulation among this population (Rochat et al., 2013), which may reduce the potential for the recovery of cognition and activities of daily living (Mikami et al., 2013). Moreover, traditional cognitive training approaches can suffer from poor protocol adherence (Rebok et al., 2007).

Competition, challenge, and working with peers have been proposed to increase intrinsic motivation in educational environments (Eastern, 2009). Social interactions, with a strong preference for competitive over collaborative paradigms (Schmierbach et al., 2012), can also promote fun and improve the subjective experience (Gajadhar et al., 2008). In rehabilitation, as in other fields, social interactions derived from group interventions can positively influence not only the experience but also the performance of individuals (Baur et al., 2018). Additionally, the inclusion of competitive group dynamics in rehabilitative interventions has also been shown to increase commitment and involvement in the intervention tasks compared with other game modalities (Ede et al., 2015). However, the perception and enjoyment of a competitive interaction can be modulated by the personal traits of participants (Song et al., 2013). Competition is likely to be appreciated by competitive individuals, whereas it could have detrimental effects for less competitive subjects (Song et al., 2013). These results are in accordance with the reports of healthy subjects and a small group of chronic subjects post-stroke, which suggested that rehabilitation sessions were more enjoyable when working with others than when working individually, with subjects showing preference for either competitive or non-competitive interactions according to their individual traits (Novak et al., 2014).

Despite the consistent predilection for group interventions, most studies examining the rehabilitation of cognitive impairments post-stroke, including those focused on attention, enroll subjects in individual interventions. The effects of competition on the effectiveness and motivation of group interventions aimed at improving attention deficits post-stroke, therefore, remains unexplored, although competition-based interventions could potentially increase adherence to treatment. We hypothesized that a specifically designed group intervention, combining conventional and technological tools that are administered in a competitive manner, would be more effective and motivating than an intervention that utilized the same tools, administered in a non-competitive manner, for the rehabilitation of attention deficits following stroke; however, the effectiveness of such an intervention could also depend on personal preferences. Consequently, this study aimed to determine the effectiveness of a competitive intervention to improve attention and motivation, compared with a non-competitive alternative, and to determine the moderating effects of individual competitiveness on these factors, in a group of individuals post-stroke.

## Materials and Methods

### Participants

Participants were recruited from the long-term neurorehabilitation programs of three neurorehabilitation units: Hospital Vithas Valencia al Mar (València, Spain), the Brain Injury Centre Vithas Vinalopó (Elx, Spain), and Hospital Vithas Sevilla Aljarafe (Sevilla, Spain). The inclusion criteria for participation in the study were as follows: time since injury > 6 months; impaired attentional skills that could potentially benefit from a specific intervention, as determined by a total score below the age-corrected normative value on the d2 Test of Attention (Brickenkamp, 2002); fairly good cognitive condition, as determined by a score > 23 on the Mini-Mental State Examination (Folstein et al., 1975); the ability to read and write; and inclusion in a conventional cognitive rehabilitation program for at least 3 months in one of the recruiting centers. Participants were excluded for the following: impaired comprehension that would hinder the sufficient understanding of instructions, as determined by a score below 45 on the Mississippi Aphasia Screening Test (Romero et al., 2012); severe paresis of the upper limb that would prevent interactions with the intervention instruments, as defined by a Brunnstrom Approach classification above stage 3 (Shah, 2010); spatial neglect, as defined by a score below 129 on the Behavioral Inattention Test (Wilson et al., 1987); emotional or behavioral circumstances that would impede adequate collaboration, as defined by a score above 4 on the Neuropsychiatric Inventory (Wood et al., 2000); severe visual impairments that, in the judgement of the assigned therapist of each subject, would not allow interaction with the instruments; and participation in any non-conventional cognitive programs, such as non-invasive brain stimulation interventions, prior to the intervention.

Participants were randomly assigned to either a non-competitive or a competitive intervention group. The randomization schedule was computer-generated, using a basic random number generator, at a ratio of 1:1. The allocation sequence was generated by an independent researcher and concealed from the study administrators. A sealed envelope was given to the coordinators of the neuropsychology departments to identify the group for each participant. The therapists who performed the assessments and the researchers who performed the data analysis were blinded to each participant's allocated intervention. The neuropsychologists who conducted the intervention, in contrast, could not be blinded to the group allocation.

A minimum sample size of forty-four participants was estimated to achieve an alpha of 0.05, a statistical power of 0.95, and an effect size of 0.25 while allowing for a dropout rate of 20%.

This study was registered at clinicaltrials.gov (NCT02220816) and was approved by the Institutional Review Board of the Hospital Vithas Valencia al Mar (NI116282DAV0/3). All participants provided written informed consent before enrollment.

### Instrumentation

Both conventional exercises and interactive computerized multi-touch exercises were specifically designed to train processing speed and sustained, selective, and divided attention, while simultaneously involving working memory and inhibition.

The conventional exercises included paper-and-pencil tasks, based on cancellation, choice-making, spot-the-difference, order and sequencing, series completion, connect-the-dots, odd-one-out, and missing character exercises, which required attentional and other cognitive skills. The level of difficulty for these exercises could be configured by adjusting the numbers and types of stimuli and distractors, the lengths of the sequences and series, and the complexity of the illustrations. A digital countdown timer displayed the time remaining during exercise performance. After completion, a therapist provided performance feedback and reported the time taken to complete the exercises to each participant, individually, in the non-competitive intervention, or to all participants, in the competitive intervention, which included information regarding each participant's rank, relative to the performance of the other participants.

Interactive computerized multi-touch exercises were developed, including eight different games that featured go/no-go, timed multi-choice, and cancellation tasks, framed as different sports, Olympic events, and scenarios, with each game focusing on a specific combination of attentional and other cognitive skills (Table 1). In addition to the cognitive demands of each game, the timing of the required actions was paramount. During the marathon and public games, participants were required to select the correct choice and to identify a target among distractors, respectively, within the shortest possible time. In contrast, all actions in the cycling, tennis, duathlon, and triathlon games had to be performed with precise timing, which was indicated in the game with changing colors (for instance, in the cycling game, when an obstacle entered the area of interaction for the character, the area turned green, indicating that the user should press a button at that moment to avoid the obstacle). This approach trained both processing speed and inhibition. The level of difficulty for each game could be configured by adjusting a group of parameters (Table 1). The games were displayed in four separate areas of interaction, with each area corresponding to and oriented relative to a different side of a multi-touch table system (Table 1). All games, except the public game, featured cartoon-like characters playing sports that the users controlled by touching virtual buttons, with the aim of achieving the best possible performance in the virtual event. During the public game, in contrast, users were required to search and identify a series of target elements among spectators at a sporting event, by touching the elements on the virtual scene. All interactive elements had considerable size, to allow for the participation of individuals with moderately impaired arm-hand coordination and spasticity.

**Table 1:** Description of the interactive computerized multi-touch exercises.

**Exercise**	**Skill**	**Environment**	**Interaction**	**Objective**	**Input parameters**	**Output parameters**	**In-game screenshot**
Marathon	Sustained attention	A road with a runner. Different items appear above the runner at different speeds.	One button: • To pick up items	To pick up water and fruits as fast as possible, without picking up the bricks	Speed	Correct answers, omissions, commissions	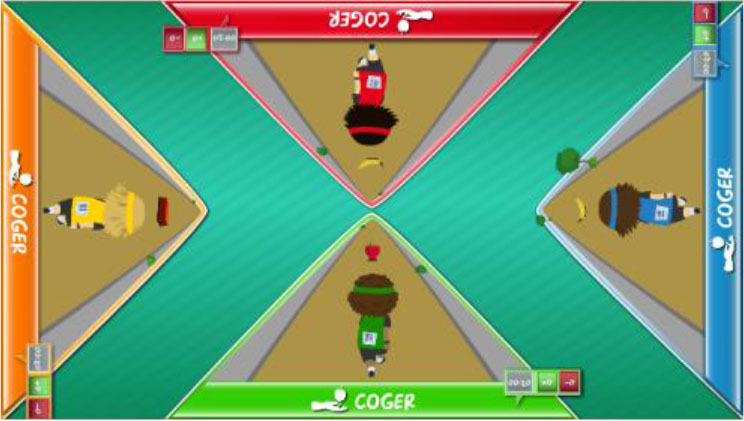
Cycling	Selective attention	A road with a cyclist. Different obstacles approach the cyclist	Two buttons: • To turn sideways • To brake	To avoid puddles and logs on the road and to stop at level crossings	Speed, size of area of interaction	Correct answers, omissions, commissions	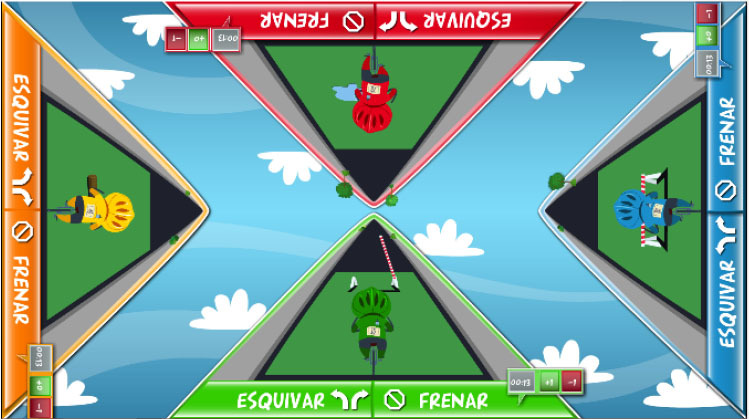
Tennis	Processing speed, inhibition	A doubles game on a tennis court	Two buttons: • Left player hits • Right player hits	To return the ball with the left or right player, as appropriate	Speed, size of area of interaction, time between ball shots	Correct answers, omissions, commissions	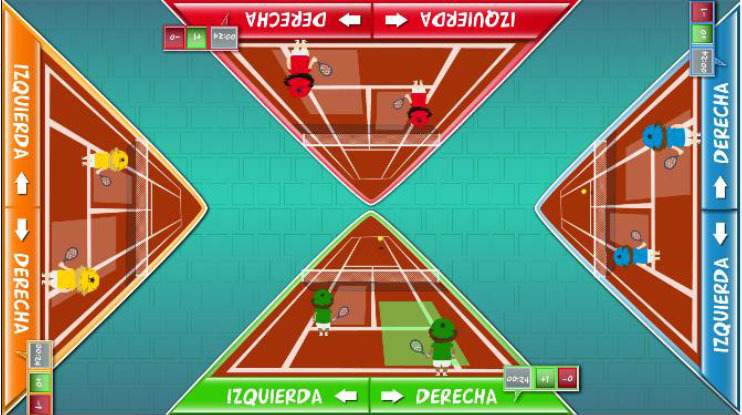
Public	Processing speed, selective attention	A crowd of spectators in a grandstand	Screen touches on the items to be found	To identify facial features and pieces of clothing in the crowd as fast as possible. In the competitive mode, the same scenario is shown to all users, and the items disappear when they are found by any user. Users must identify the elements before other users.	Time to identify the items, number of characters, number of items to be found	Correct answers, omissions, commissions	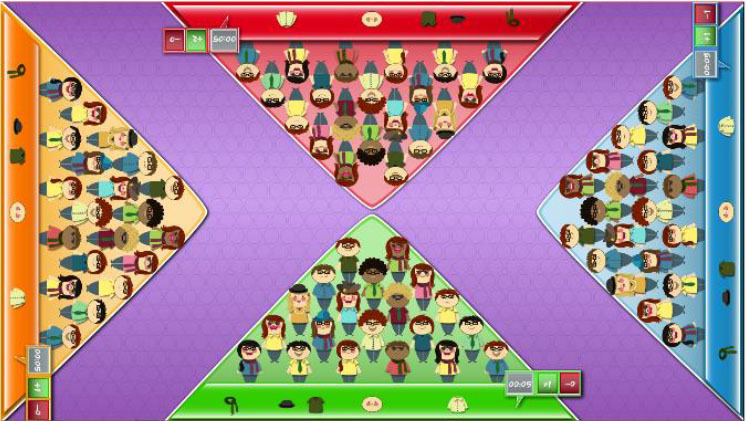
Football	Selective and divided attention	A football field featuring players on two different teams	Screen touches on the players	To identify football players who have previously been highlighted and a ball after a play	Number of players in the field, number of players to track, duration of play, time to answer, presence of distractor (a ball)	Correct answers, omissions, commissions	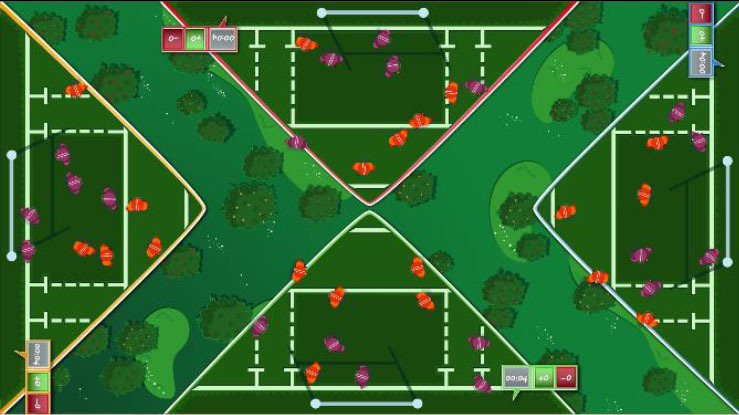
Soccer	Selective attention, working memory	A soccer field with players on two teams	Screen touches on the players	To connect dots, repeating a previously displayed sequence of ball passes, forwards or backwards. The displayed sequences are increased by one pass when correct answers are provided	Time to identify a dot, number of correct answers needed to increase the sequence	Correct and incorrect answers	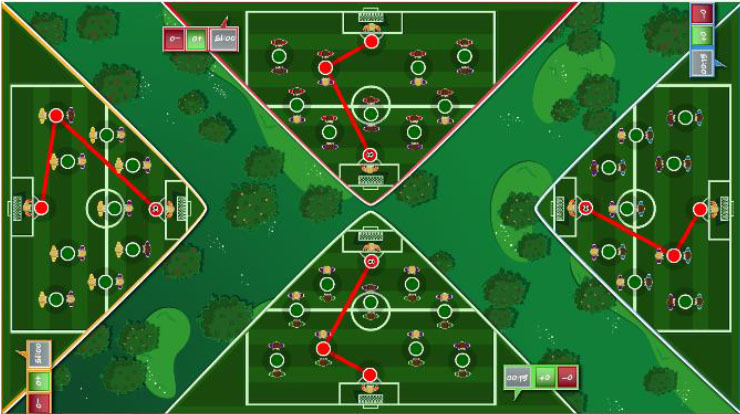
Duathlon	Divided attention	A split screen, displaying a marathon and a cycling event, with the respective athletes	Two buttons: • To pick up items • To turn sideways	To pick up water and fruits (marathon) and avoid puddles and logs (cyclist)	Marathon: • Time to pick up the items Cycling: • Speed, size of area of interaction	Correct and incorrect answers, for each event	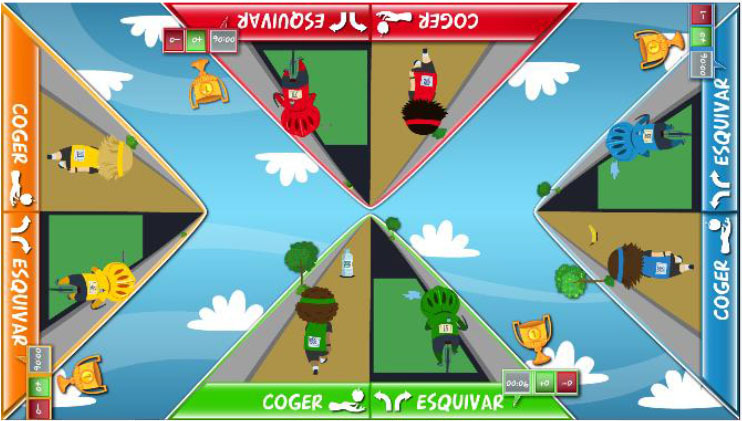
Triathlon	Divided attention	A split screen, displaying a marathon, a cycling event, and a swimming event, with the respective athletes	Three buttons: • To pick up items • To turn sideways • To flip turn	To pick up water and fruits (marathon), avoid puddles and logs (cyclist), and execute flip turns (swimmer)	Marathon: • Time to pick up the items Cycling: • Speed, size of area of interaction Swimming: • Speed, size of area of interaction	Correct and incorrect answers, for each event	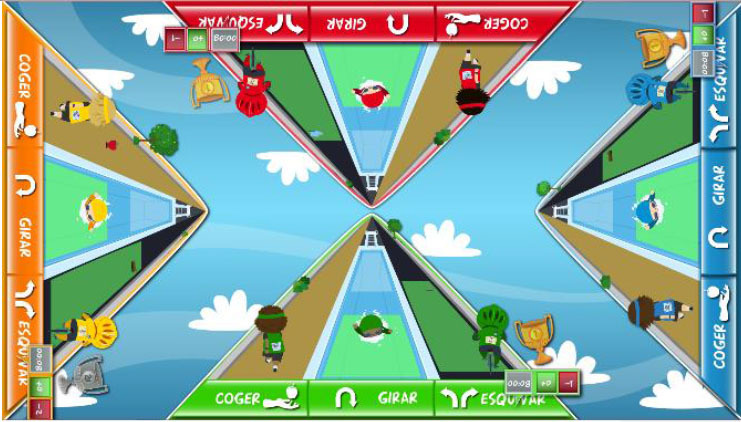

**Figure 1:**
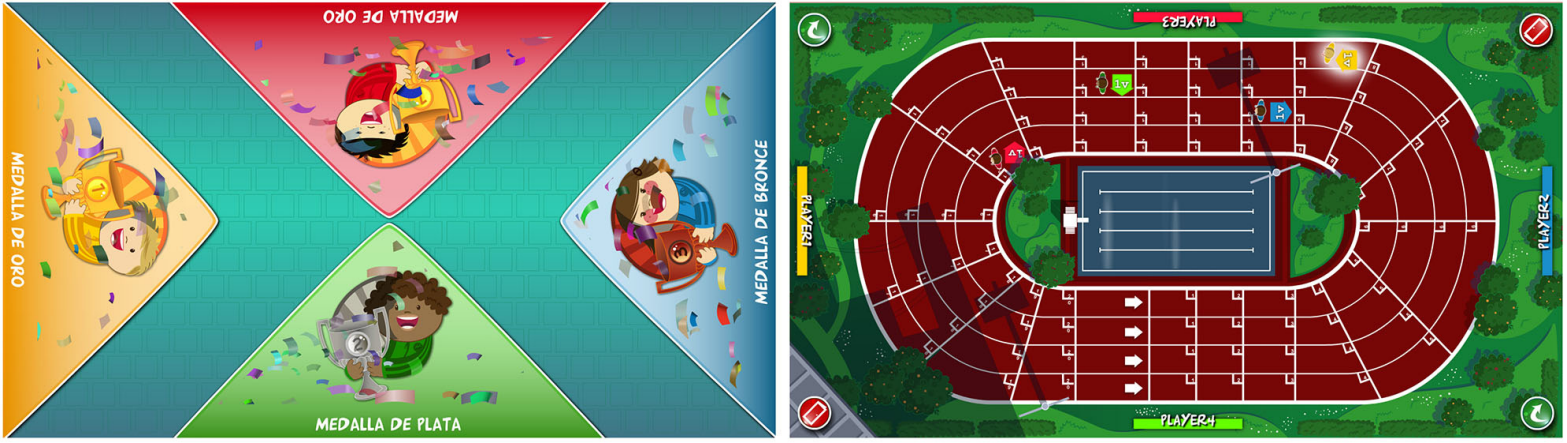
Feedback provided by the computerized multi-touch exercises in the competitive mode. After each competitive exercise, the system provided feedback of the participants' performance, using a virtual podium ceremony (left), and current position in the session, using an athletics track (right).

Points were awarded to each user, according to individual performance, at the end of each game. The system provided information regarding the time remaining, as well as extrinsic feedback regarding performance, during the games, such as the number of correct responses and mistakes, and a score screen summarized the performance after each game. In the competitive mode, the extrinsic feedback provided during the games included the provisional rank relative to other participants. Exclusive to competitive mode, after each game, the system displayed a virtual podium ceremony, followed by an image of a running track, featuring athlete-like characters that represented the users. The characters moved forwards on the track according to their performances during the exercises (Figure 1). Specifically, the winner moved four steps forward, the runner-up moved three steps forward, and so on. In the case of a draw, the users achieved the same score and, consequently, moved the same number of steps. This running track provided visual feedback regarding their current rankings during each session.

All game interactions were facilitated using a multi-touch table system. The system consisted of a conventional 42″ LCD screen that was embedded in a conventional table and oriented in a horizontal plane, parallel to the floor, which provided visual and auditory feedback. A multi-touch frame, which was fixed over and along the screen provided interactive capability, enabling the detection of up to 32 simultaneous finger touches. A group of participants could be arranged, with one person on each side of the table, and interact with the multi-touch table system, allowing group-based interventions to be performed, with a high degree of participant-reported usability and motivation (Llorens et al., 2015).

**Figure 2:**
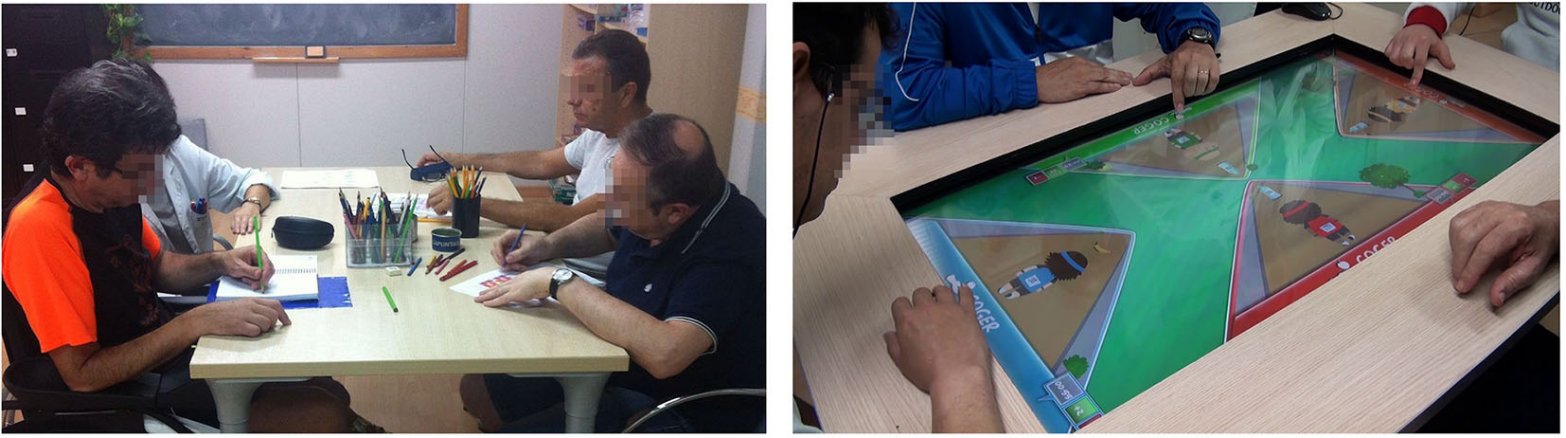
Experimental setting using conventional and interactive computerized multi-touch exercises. All the participants trained in groups using conventional (left) and interactive computerized multi-touch exercises (right).

Figure 2 shows an experimental administration of both the conventional and interactive computerized exercises.

### Procedure

The intervention consisted of 20 one-hour sessions, administered in groups of three or four participants, 3 days a week. All sessions combined 30 min of conventional exercises with 30 min of interactive computerized multi-touch exercises. Each session consisted of 8 6-min exercises, with 1.5-min breaks between each exercise. Both conventional and computerized exercises were administered in counterbalanced order, in such a way that an exercise was not repeated until all other types of exercises were administered. The difficulties of all exercises, including both conventional and computerized exercises, were adjusted according to each participant's condition, which was determined in an exploratory session. All sessions were conducted by experienced neuropsychologists, who monitored, instructed, and provided feedback to the participants.

Both the non-competitive and competitive interventions were, consequently, time- and difficulty-matched and were equally administered. The only differences between the interventions were their objectives and the feedback provided. Participants in the non-competitive group performed their exercises individually, with instructions to perform to the best of their abilities (i.e., trying to finish the exercises as soon as possible, with the highest number of correct answers and the lowest number of errors), and received feedback regarding their individual performances. In contrast, participants in the competitive group competed to achieve the best performance among all competitors in the sessions, and received feedback regarding their own and other participants' performances, as described above.

All participants were assessed both before and after the intervention, using a battery of clinical tests that evaluated processing speed, sustained, selective and divided attention, working memory, and inhibition, which represent the primary abilities that were trained by both interventions (Table 2). The assessment included the Conners' Continuous Performance Test (Homack and Riccio, 2006), the d2 Test of Attention (Brickenkamp, 2002), the Color Trail Test (D'Elia et al., 1996), the Digit Span subtest of the Wechsler Adult Intelligence Scale-Fourth Edition (Wechsler, 2008), and the Spatial Span subtest of the Wechsler Memory Scale-Fourth Edition (Wechsler, 2009).

**Table 2:** Cognitive abilities addressed by the assessment instruments.

**Cognitive ability**	**Measures**
Processing speed	• Conners' Continuous Performance Test—Reaction Time • d2 Test of Attention—Total Score • Color Trail Test—Part A
Sustained attention	• Conners' Continuous Performance Test—Omissions • d2 Test of Attention—Total Score
Selective attention	• d2 Test of Attention—Total Score • Color Trail Test—Part A • Digit Span • Spatial Span
Divided attention	• Color Trail Test—Part B
Working memory	• Digit Span • Spatial Span
Inhibition	• Conners' Continuous Performance Test—Commissions • Conners' Continuous Performance Test—Perseveration • d2 Test of Attention—Total Score

In addition, the subjective experiences for both interventions were assessed based on self-reported measures of interest/enjoyment, perceived competence, pressure/tension, and value/usefulness, using the Intrinsic Motivation Inventory (McAuley et al., 1989). The competitiveness of each participant was also assessed, after the intervention, using the Revised Competitiveness Index (Houston et al., 2002).

### Data Analysis

The comparability of both groups at baseline was investigated with independent samples Student's *t-*tests and chi-square or Fisher's exact tests, as appropriate. Mixed factorial analyses of variance (ANOVAs), with time (before and after treatment) as the within-subjects factor and treatment option (competitive vs. non-competitive) as the between-subjects factor, were performed, for all cognitive and motivational measures. ANOVA findings that violated the sphericity assumption were accommodated by the Greenhouse-Geisser conservative degrees of freedom adjustment. The main effects of time and treatment option and the time-treatment option interaction effects were evaluated. Partial eta squared ($\eta_{\text{p}}^{2}$) was computed for each ANOVA, as a measure of the effect size. Effect size values may range from 0 to 1, with higher values representing higher proportions of variance that can be explained by the independent variable. Finally, moderation analyses were performed, to examine whether the effects of group allocation on the clinical effectiveness and motivation associated with the intervention were moderated by the competitiveness of the participants. Analyses were performed using the procedure described by Hayes (Hays, 2018), with the macro PROCESS (version 3.3). In these analyses, the competitive group was coded as “1,” and the non-competitive group was coded as “2.” Baseline scores for all clinical variables were entered as covariates of the dependent variables, in each model. Tests of significance (p < 0.05) or a confidence interval (not including zero) for the interaction “group × motivation” were used to examine whether motivation moderated the effects of group allocations on post-treatment scores for all clinical and subjective measures.

The α level was set at 0.05 for all analyses (two-sided).

## Results

### Participants

**Figure 3:**
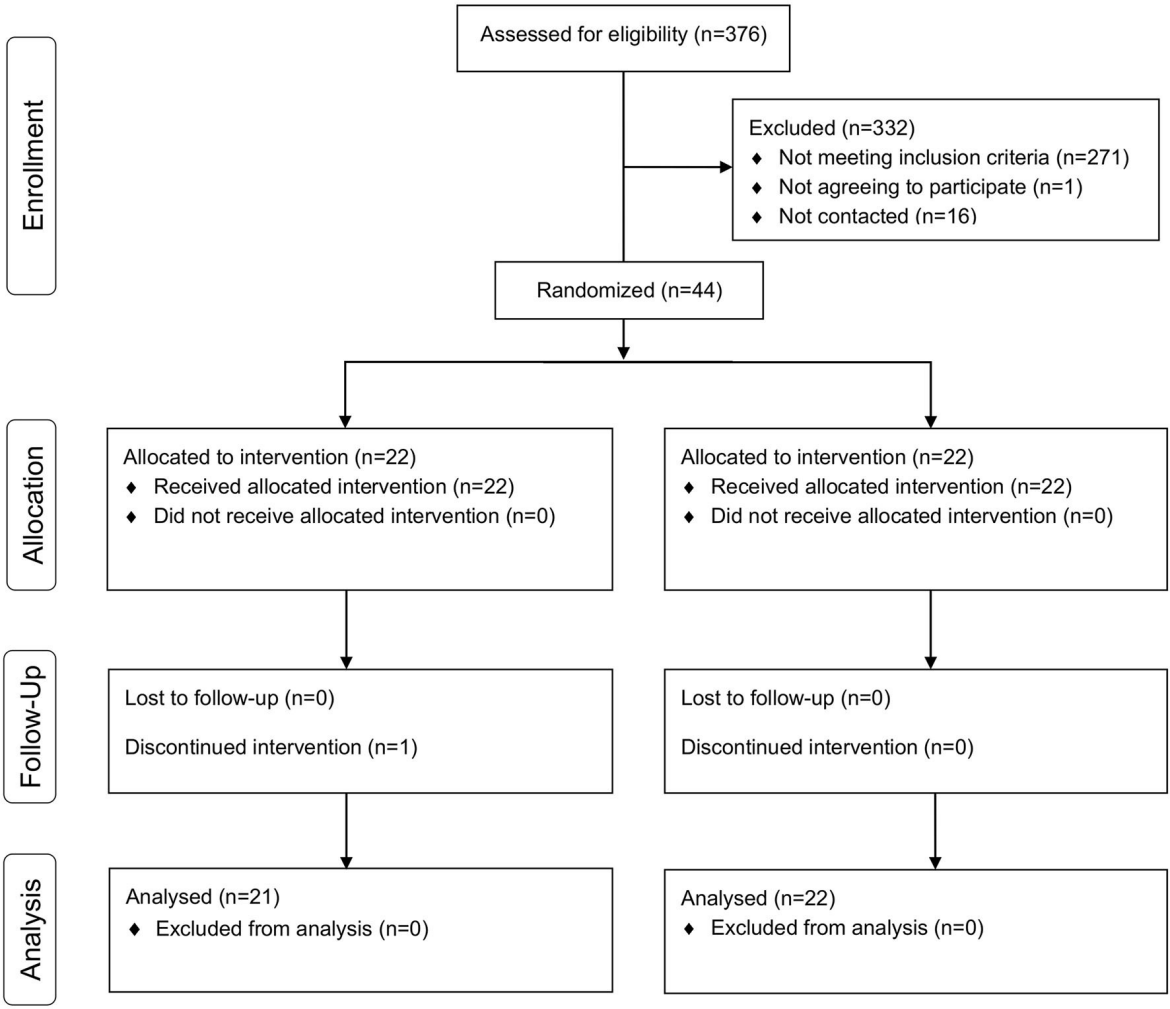
CONSORT flow diagram. Progress through the phases of the parallel randomized trial of both groups.

During the recruitment period, a total of 376 individuals were identified who were attending a long-term neurorehabilitation program at one of the recruiting centers (Figure 3), among which, 61 (16.2%) participants met the criteria for study participation. Forty-four subjects were randomly approached and agreed to participate in the trial. These participants were randomized into the non-competitive and competitive groups, and were grouped in groups of three or four in each recruiting center. One participant in the non-competitive group was discharged and dropped out of the study; consequently, her data was not included in the final analysis. All included participants attended all sessions.

The final sample included 43 participants, 19 women and 24 men, with a mean age of 52.3 ± 14.8 years (Table 3). Participants experienced either an ischemic (*n* = 21) or hemorrhagic stroke (*n* = 22) in the left (*n* = 13), right (*n* = 16) or both hemispheres (*n* = 7), or other brain region (*n* = 7), with a mean time since injury of 403.3 ± 243.2 days (Table 3) (see Supplementary Materials for individual information of each participant and further details about neuropathological and pharmacologic information). Both groups were comparable in all demographic, personality, and clinical variables.

**Table 3:** Characteristics of the participants.

	**Non-competitive group (*n* = 21)**	**Competitive group (*n* = 22)**	**Significance**
Sex (*n*, %)			NS (*p* = 0.543)
Women	8 (38.1%)	11 (50.0%)	
Men	13 (61.9%)	11 (50.0%)	
Age (years)	52.9 ± 10.6	51.7 ± 18.1	NS (*p* = 0.805)
Etiology (*n*, %)			NS (*p* = 0.366)
Ischemic stroke	12 (57.1%)	9 (40.9%)	
Hemorrhagic stroke	9 (42.9%)	13 (59.1%)	
Oxford classification^a^ (*n*, %)			NS (*p* = 0.613)
TACI	2 (16.7%)	0 (0%)	
PACI	5 (41.7%)	5 (55.6%)	
LACI	2 (16.7%)	2 (22.2%)	
POCI	3 (25.0%)	2 (22.2%)	
Lesion side (*n*, %)			NS (*p* = 0.378)
Left	7 (33.3%)	6 (27.3%)	
Right	9 (42.9%)	7 (31.8%)	
Bilateral	1 (4.8%)	6 (27.3%)	
Brainstem	2 (9.5%)	2 (9.1%)	
Cerebellum	2 (9.5%)	1 (4.5%)	
Time since injury (months)	433.6 ± 258.5	374.3 ± 229.9	NS (*p* = 0.431)
Education (years)	12.9 ± 4.3	11.0 ± 4.0	NS (*p* = 0.148)
Mini-Mental State Examination [0–30]	26.8 ± 1.8	26.3 ± 1.7	NS (*p* = 0.367)
Mississippi Aphasia Screening Test [0–50]	48.6 ± 1.5	48.0 ± 1.6	NS (*p* = 0.235)
D2 Test of Attention. Total score	228.6 ± 42.8	207.6 ± 68.8	NS (*p* = 0.240)
Competitiveness [9–45]	36.1 ± 8.0	33.0 ± 13.1	NS (*p* = 0.351)

*Sex, etiology, and lesion side are expressed as a percentage of the total number of participants. Age, time since injury, and scores in the clinical and personality measures are expressed in terms of mean and standard deviation. NS, non-significant*.

a *In accordance with the Oxfordshire Community Stroke Project classification*.

### Clinical Effectiveness

A significant time effect was detected for all measures of cognitive function (Table 4) (see Supplementary Materials for further details about the individual progression on the d2 Test of Attention). However, the competitive group showed significantly greater improvements in all cognitive abilities except divided attention, compared with the non-competitive group. Specifically, improvements in processing speed, selective attention, and working memory were demonstrated by all cognitive measures used to evaluate these skills. The larger effects of the competitive intervention for improving sustained attention and inhibition, however, were supported by scores in the d2 Test of Attention but not by measures of Omissions, Commissions, or Perseverations on the Conners' Continuous Performance Test. Although no significant differences between groups emerged for divided attention, the results for Part B of the Color Trail Test showed improvements after the competitive intervention, compared with the non-competitive intervention, which tended toward significance (*p* = 0.054).

**Table 4:** Treatment effects on cognitive function.

	**Initial assessment**	**Final assessment**	**Change**	**Significance**
**CONNERS' CONTINUOUS PERFORMANCE TEST**				
Reaction time (ms)				T^**^(*p* < 0.001, $\eta_{\text{p}}^{2}$ = 0.29)
Non-competitive group	478.5 ± 103.1	468.6 ± 93.6	−9.9 ± 38.8	GxT^**^(*p* = 0.006, $\eta_{\text{p}}^{2}$ = 0.17)
Competitive group	513.1 ± 111.0	457.4 ± 90.4	−55.6 ± 61.9	
Omissions (n)				T^*^(*p* = 0.049, $\eta_{\text{p}}^{2}$ = 0.09)
Non-competitive group	11.2 ± 17.5	8.1 ± 9.8	−3.1 ± 10.3	GxT(*p* = 0.714, $\eta_{\text{p}}^{2}$ < 0.01)
Competitive group	13.8 ± 19.5	9.3 ± 15.5	−4.5 ± 14.0	
Commissions (n)				T^*^(*p* = 0.036, $\eta_{\text{p}}^{2}$ = 0.10)
Non-competitive group	10.9 ± 5.0	9.6 ± 5.4	−1.2 ± 5.0	GxT (*p* = 0.683, $\eta_{\text{p}}^{2}$ < 0.01)
Competitive group	10.9 ± 7.4	9.1 ± 5.3	−1.8 ± 4.3	
Perseverations (n)				T^*^(*p* = 0.028, $\eta_{\text{p}}^{2}$ = 0.11)
Non-competitive group	1.7 ± 2.9	1.2 ± 1.7	−0.5 ± 1.8	GxT(*p* = 0.246, $\eta_{\text{p}}^{2}$ = 0.03)
Competitive group	3.3 ± 5.9	1.8 ± 3.1	−1.5 ± 3.6	
**D2 TEST OF ATTENTION**				
Total score				T^**^(*p* < 0.001, $\eta_{\text{p}}^{2}$ = 0.31)
Non-competitive group	228.6 ± 42.9	257.8 ± 63.5	29.2 ± 54.4	GxT^*^(*p* = 0.038, $\eta_{\text{p}}^{2}$ = 0.10)
Competitive group	207.6 ± 68.8	294.4 ± 117.5	86.8 ± 111.2	
**COLOR TRAIL TEST**				
Part A (s)				T^*^(*p* = 0.015, $\eta_{\text{p}}^{2}$ = 0.14)
Non-competitive group	70.1 ± 30.4	68.4 ± 32.8	−1.7 ± 20.5	GxT^*^(*p* = 0.036, $\eta_{\text{p}}^{2}$ = 0.10)
Competitive group	80.1 ± 59.2	59.2 ± 22.9	−20.9 ± 35.4	
Part B (s)				T^**^(*p* < 0.001, $\eta_{\text{p}}^{2}$ = 0.23)
Non-competitive group	145.1 ± 48.8	135.4 ± 52.0	−9.7 ± 28.0	GxT(*p* = 0.054, $\eta_{\text{p}}^{2}$ = 0.09)
Competitive group	179.4 ± 84.5	144.9 ± 48.3	−20.9 ± 35.5	
Digit span (n)				T^**^(*p* = 0.002, $\eta_{\text{p}}^{2}$ = 0.22)
Non-competitive group	12.3 ± 3.1	12.7 ± 4.0	0.4 ± 2.2	GxT^*^(*p* = 0.037, $\eta_{\text{p}}^{2}$ = 0.10)
Competitive group	10.9 ± 2.7	12.7 ± 2.4	1.8 ± 1.9	
Spatial span (n)				T^**^(*p* < 0.001, $\eta_{\text{p}}^{2}$ = 0.29)
Non-competitive group	13.1 ± 3.5	13.9 ± 3.4	0.8 ± 2.1	GxT^*^(*p* = 0.038, $\eta_{\text{p}}^{2}$ = 0.10)
Competitive group	12.0 ± 3.2	14.5 ± 2.9	2.4 ± 2.8	

*Clinical data are given in terms of mean and standard deviation. T, time effect; GxT, group by time effect*.

* *p < 0.05*,

** *p < 0.01*.

### Motivation

Participants in the competitive group reported greater enjoyment than their non-competitive peers (*p* = 0.026) (Table 5). However, no significant differences were found in perceived competence, pressure/tension, or value/usefulness, although the competitive group provided better scores for these items.

**Table 5:** Subjective experience elicited by the treatment interventions.

	**Non-competitive group**	**Competitive group**	**Significance**
Intrinsic Motivation Inventory			
Interest/enjoyment [1–7]	5.1 ± 1.0	5.8 ± 0.8	*p* = 0.026
Perceived competence [1–7]	4.9 ± 1.1	5.3 ± 1.3	NS (*p* = 0.344)
Pressure/tension^a^ [1–7]	2.6 ± 1.5	2.2 ± 1.3	NS (*p* = 0.359)
Value/usefulness [1–7]	5.3 ± 1.1	5.7 ± 1.2	NS (*p* = 0.289)

*Data are given in terms of mean and standard deviation. NS, non-significant*.

a *results to this item should be interpreted opposed to all other items, as low scores are associated to a better characteristic*.

### Influence of Competitiveness on Clinical Effectiveness and Motivation

Competitiveness did not moderate the effects of group allocation on the clinical effectiveness of the intervention, as the interaction between competitiveness and group allocation was not significant in the moderation analyses. Specifically, the moderation analyses showed no moderating effects of competitiveness for reaction time [F_(1, 38)_ = 2.49, *p* = 0.123], omissions [F_(1, 38)_ = 0.01, *p* = 0.927], commissions [F_(1, 38)_ = 0.38, *p* = 0.540], or perseverations [F_(1, 38)_ = 1.84, *p* = 0.183] on the Conners' Continuous Performance Test, the total score of the d2 test of attention [F_(1, 38)_ = 1.08, *p* = 0.306)], Part A [F_(1, 38)_ = 2.23, *p* = 0.143] or Part B [F_(1, 38)_ = 3.86, *p* = 0.057] of the Color Trail Test, the Digit Span [F_(1, 37)_ = 0.02, *p* = 0.899], or the Spatial Span [F_(1, 38)_ = 0.40, *p* = 0.530]. Competitiveness also showed no moderating effects on group allocation for the subjective impressions elicited by the interventions, according to the results of the analyses on the interest/enjoyment [F_(1, 39)_ = 1.30, *p* = 0.261], perceived competence [F_(1, 39)_ = 1.46, *p* = 0.234], pressure/tension [F_(1, 39)_ = 0.09, *p* = 0.771], or value/usefulness [F_(1, 39)_ = 1.47, *p* = 0.232], assessed by the Intrinsic Motivation Inventory.

## Discussion

This study investigated the effectiveness and motivation of a group-based intervention, combining conventional and computerized multi-touch exercises, when administered in either a competitive or non-competitive manner, on attention deficits post-stroke, and also examined the moderating effects of individual competitiveness on these variables. The results showed that the competitive intervention provided greater improvements for all cognitive abilities, except for divided attention, and participants reported greater enjoyment, regardless of individual preferences for competition.

Although the great diversity of outcome measures between studies restrict a detailed comparison of our results with those of previous investigations, the effects of both the competitive and non-competitive interventions on attention deficits agree with the existing reports. First, the observed decrease in the reaction time, assessed by Conners' Continuous Performance Test, indicated an improved response time to stimuli, which is commonly slowed after stroke (Alonso-Prieto et al., 2002). Similar results were reported for the training effects in previous studies, which were measured using the Test of Attentional Performance (Röhring et al., 2004) and the Vienna Test System (Sturm and Willmes, 1991). Second, the results of the d2 Test of Attention, which is commonly used to assess selective and sustained attention, were also supported by previous findings using this test (Sturm and Willmes, 1991; Röhring et al., 2004). Third, the improvements in the Color Trail Test, a language-free version of the Trail Making Test, are consistent with the effects of earlier cognitive interventions designed to improve sustained and divided attention, which were measured using the latter test (Barker-Collo et al., 2009; Winkens et al., 2009; Yoo et al., 2015; Faria et al., 2016). Finally, the enhancements in selective attention and working memory observed following our intervention are supported by the results of previous interventions, which showed improvements in cognitive functioning based on both the Digit Span and Spatial Span (Yoo et al., 2015; das Nair et al., 2016). All of these results support the reliability of intensive, specific programs for improving cognitive impairment after stroke, including, but not limited, to attention deficits. However, improvements in attention could be especially relevant, as improved attention could facilitate the rehabilitation of other cognitive skills and maximize functional recovery (Hyndman et al., 2008). The absence of more detailed neuropathological information of the brain lesions prevented further investigation of the effects of location of stroke-related brain lesions on the effectiveness of the interventions.

The greater improvements demonstrated by participants in the competitive group, in almost all attentional domains, could be explained by the increased effort of participants compared with those in the non-competitive group, which could potentially be promoted by the anxiety-inducing factors derived from competition, such as social evaluation (Cooke et al., 2013). The increased benefits of competition observed in this study are in line with previous reports on motor function (Baur et al., 2018; Mandehgary Najafabadi et al., 2019), physical effort, and intensity (Le Bouc and Pessiglione, 2013; Goršič et al., 2017). The increased effects of competition could be detected in almost all time-dependent measures. Improvements in the response time and the processing speed, demonstrated by a decrease in the Reaction Time, assessed by the Conners' Continuous Performance Test, could positively contribute to a reduction in the time necessary to perform other tests, such as the Color Trail Test, or to the improved processing of multiple stimuli during a given time, as in the d2 Test of Attention. In line with this, improvements in the speed of performance have previously been reported after a specific intervention designed to improve attention compared with usual care (Winkens et al., 2009). Competitive strategies that challenge processing speed could be especially interesting to improve this ability, which has been shown to be pronouncedly impaired after stroke (Rasquin et al., 2004; Su et al., 2015), especially after right-sided lesions (Gerritsen et al., 2003).

The absence of differences between groups for the other measures of the Continuous Performance Test, other than Reaction Time, may demonstrate that competitive dynamics are not specifically beneficial for inhibition. However, among all other measures, only the Reaction Time of the Continuous Performance Test has been shown to have satisfactory test-retest reliability in individuals with chronic stroke (Chen et al., 2009). In addition, behavioral measures of response inhibition, such as stop-signal tasks or go/no-go tasks, which are assessed by the Continuous Performance Test, have a weak relationship with self-reported impulsivity (Sharma et al., 2014) and are likely to engage more than a single underlying process (Skippen et al., 2019).

The significant decrease observed in the time to complete part A of the Color Trail Test, but not part B, after the competitive intervention compared with the non-competitive intervention could be due to the different skills required for both parts. As in the Trail Making Test, part A of the Color Trail Test predominantly measures processing speed, which could explain sensitivity to the effects of the intervention, whereas part B has been suggested to be a measure of cognitive flexibility (Kopp et al., 2015). However, although no significant differences were detected between the competitive and non-competitive groups for part B, differences were observed that tended toward significance, and significant differences may emerge if larger samples or longer interventions had been considered. The effects of a competitive strategy compared with other cognitive interventions on performance in the trail tests (Barker-Collo et al., 2009; Faria et al., 2016) should, therefore, be considered. The inclusion of part B of the Color Trail Test as the only measure of divided attention may have hindered the more accurate detection of training effects on this attentional ability. Although this subtest includes a large number of stimuli that must be attended, almost twice the number of its counterpart in the Trail Making Test, performance on this test may be modulated by other cognitive skills, such as cognitive flexibility, in addition to the specific ability to attend to two tasks simultaneously. Although this test has been used by previous reports to assess divided attention (Barker-Collo et al., 2009; Winkens et al., 2009), other measures that imply attending to two simultaneous-choice reaction-time tasks, such as the Test for Attentional Performance (Zimmermann et al., 2004), may better reflect the effects of the intervention, which could also explain the differences observed between the present study and previous studies that have reported improved divided attention after cognitive rehabilitation programs in stroke survivors (Virk et al., 2015; Loetscher et al., 2019).

The significant improvements observed in both the Digit Span and Spatial Span Tests after the competitive intervention, compared with the non-competitive paradigm, indicated the positive effects of competition on working memory. Although some controversy exists regarding the mechanisms involved in both forward and backward variations of these tests (Donolato et al., 2017) and the differences between tests (Wilde and Strauss, 2002), both tests are generally accepted to encompass working memory and engage executive control, especially in the backward condition (Wilde et al., 2004). Importantly, these skills have been associated with rehabilitation participation (Skidmore et al., 2010) and have been shown to be cognitive predictors of social function (Hommel et al., 2009).

The subjective experiences elicited by both interventions support the acceptance of the combination of conventional and interactive computerized multi-touch exercises, which agrees with previous studies examining the rehabilitation of cognitive (Llorens et al., 2015) and motor impairments (Colomer et al., 2016). The increased enjoyment experienced during the competitive intervention should, therefore, be highlighted. This finding is also in line with previous studies, which rated a competitive interaction as being more enjoyable than other alternatives (Walker, 2010; Goršič et al., 2017). Furthermore, a previous investigation examining the subjective experiences of different interactive modalities within a rehabilitation setting, using the Intrinsic Motivation Inventory, also failed to identify differences in dimensions other than interest/enjoyment (Goršič et al., 2017). Interestingly, despite the name of the assessment instrument, the interest/enjoyment subscale is considered to be the only measure of intrinsic motivation, *per se*, included in the questionnaire (Intrinsic Motivation Inventory, 1994). Higher scores on this subscale could also explain the larger training effects observed for participants in the competitive group, as increased enjoyment has been shown to mediate improved performance (van Lange, 2006; Cooke et al., 2013), which has been suggested to be associated with increased effort (Harackiewicz and Sansone, 1991; Ryan and Deci, 2000; Cooke et al., 2013). Importantly, this is far from being an unidentified factor of rehabilitation post-stroke, where better rehabilitation outcomes are known to be positively associated with higher motivation during post-stroke interventions (Maclean et al., 2002), which can be partially explained by the higher adherence to treatment among individuals who are more motivated (Maclean et al., 2002; Barzel et al., 2015). Therefore, the inclusion of computerized exercises in therapeutic interventions designed for older adults, such as those used in our study, should consider that previous computer use (Turunen et al., 2019), rather than age (Lam et al., 2015), might be a determining factor for adherence. The high adherence observed in our study, with all participants attending all sessions, may have been facilitated by previous participation in a cognitive rehabilitation program at the same clinical facilities, prior to the intervention.

The lack of any mediating effects for competitiveness on either the effectiveness or motivation contradicts previous results (Song et al., 2010; Schmierbach et al., 2012; Novak et al., 2014) and our initial hypothesis. Different factors may have contributed to this finding. First, the adjustment of the level of difficulty to accommodate each particular case in our study ensured that participants could accomplish all of their objectives, which may have prevented them from being worried about worse performance and losing or disappointing their competitors, factors that have been reported to contribute to the disapproval of competition (Novak et al., 2014). Second, all participants in our study knew each other. Interestingly, player relationships have been shown to influence commitment to a task and preference for competition (Peng and Hsieh, 2012). Finally, the different methodologies used to assess the influence of competitiveness, including analyses of variance (Song et al., 2010) and covariance (Schmierbach et al., 2012), cross-validation (Novak et al., 2014), and moderation analyses, which was used in our study, could also have different sensitivities to moderating effects.

Although our intervention was exclusively focused on cognition, improvements in attention are not exclusive to cognitive training. A transference to cognitive skills has been detected after physical interventions (Zheng et al., 2016), and promising effects on cognitive function have also been reported from the combination of physical and cognitive training (Kim et al., 2011; Unibaso-Markaida et al., 2019). Interestingly, the combination of motor and cognitive training has recently been shown to provide greater improvements than either cognitive or physical training alone (Bo et al., 2019). The addition of a competitive dynamics to this combined intervention could promote further benefits.

The results of our study suggested that a group intervention that combines interactive, computerized, multi-touch exercises with paper-and-pencil tasks that are specifically designed to improve attention deficits after stroke can be effective and motivating. More importantly, effectiveness and motivation can be enhanced using a competitive strategy, without negatively affecting the subjective experience of the participants, regardless of their attitudes toward competitiveness.

## Conclusions

The addition of a competitive dynamics to a rehabilitation program designed to improve attention deficits in adults with chronic stroke provide increased benefits for both clinical effectiveness and motivation, regardless of the competitiveness of the participants, without incurring negative effects on subjective perceptions.

## Data Availability Statement

The datasets presented in this article are not readily available because confidential data is not available to share with unauthorized investigators. Requests to access the datasets should be directed to Roberto Llorens, rllorens@i3b.upv.es.

## Ethics Statement

The studies involving human participants were reviewed and approved by Hospital NISA Valencia al Mar. The patients/participants provided their written informed consent to participate in this study. Written informed consent was obtained from the individual(s) for the publication of any potentially identifiable images or data included in this article.

## Author Contributions

MN, RL, EN, and JF defined the clinical aspects of the intervention. RL, AB, and MA designed the interactive multi-touch table system and programmed the content. MN supervised the study. MN, RL, and EN participated in the original draft preparation. All authors participated in the study design. All authors contributed to the article and approved the submitted version.

## Conflict of Interest

The authors declare that the research was conducted in the absence of any commercial or financial relationships that could be construed as a potential conflict of interest.
