# Supplementary material for: Competition Enhances the Effectiveness and Motivation of Attention Rehabilitation After Stroke. A Randomized Controlled Trial
Source: Front Hum Neurosci. 2020 Sep 30;14:575403. doi: 10.3389/fnhum.2020.575403 (PMC7556305; doi:10.3389/fnhum.2020.575403)

Supplementary Material

1 Individual characteristics of the participants

Supplementary Table 1. The table provides detailed information of the individual demographic and clinical characteristics of the participants, with particular detail of the location of stroke-related brain lesion and pharmacological treatments.

								Neuropathological findings								Medication				
Participant	Group	Age	Sex	Education	Etiology	Time since injury	Lesion side	Parietal	Frontal	Cerebellum	Brainstem	Subcortical	Mini-Mental State Examination	Mississippi Aphasia Screening Test	D2 Test of Attention. Total score	Selective serotonin reuptake inhibitors	Benzodiazepines	Antiepileptics	Psychostimulant	Neuroleptics
1	NC	61	M	10	H	484	R	✓	-	-	-	-	25	49	226	✓	-	-	-	-
2	NC	48	M	21	I	716	L	✓	✓	-	-	-	28	49	229	✓	✓	✓	-	-
3	NC	38	M	11	I	608	R	✓	✓	-	-	✓	25	46	217	✓	✓	✓	-	-
4	NC	50	M	20	H	842	R	-	-	-	-	✓	25	50	212	✓	-	-	✓	-
5	NC	43	W	14	I	410	BR	-	-	-	✓	-	27	49	293	✓	✓	✓	-	-
6	NC	51	W	14	H	226	R	-	-	-	-	✓	26	48	179	✓	✓	-	✓	-
7	NC	49	M	15	I	310	C	-	-	✓	-	-	25	48	274	✓	-	✓	-	-
8	NC	54	M	10	I	1007	R	✓	✓	-	-	-	29	50	225	✓	✓	✓	-	-
9	NC	46	M	13	H	456	R	-	✓	-	-	-	28	50	294	-	-	-	-	-
10	NC	63	W	10	H	371	L	-	-	-	-	✓	27	50	148	-	-	-	-	-
11	NC	62	M	12	I	976	R	-	-	-	-	✓	25	48	303	-	-	-	-	-
12	NC	50	M	10	I	175	L	-	-	-	-	✓	26	47	223	-	-	✓	-	-
13	NC	52	W	17	I	331	R	-	-	-	-	✓	30	48	223	-	✓	-	-	-
14	NC	76	M	18	H	188	L	-	-	-	-	✓	30	50	270	✓	-	-	-	-
15	NC	48	M	8	I	464	L	-	-	-	-	✓	24	50	220	✓	-	✓	-	✓
16	NC	67	W	8	I	127	L	-	-	-	-	✓	26	50	151	✓	-	-	-	-
17	NC	67	W	4	I	341	BR	-	-	-	✓	-	26	46	193	-	✓	-	-	-
18	NC	31	W	18	H	391	BI	-	-	-	-	✓	27	49	190	✓	-	-	-	-
19	NC	43	M	14	H	231	C	-	-	✓	-	-	26	46	238	✓	-	-	-	-

20	NC	52	W	10	I	224	L	✓	✓	-	-	-	29	50	242	✓	✓	-	-	-
21	NC	59	M	14	H	228	R	-	-	-	-	✓	29	47	250	✓	-	-	-	-
22	CO	67	W	10	I	187	L	-	-	-	-	✓	24	46	127	✓	-	-	-	-
23	CO	67	W	4	I	341	BR	-	-	-	✓	-	27	47	158	-	✓	-	-	-
24	CO	71	M	8	I	225	BI	-	-	-	-	✓	25	46	175	✓	-	-	-	-
25	CO	18	M	10	I	383	L	-	-	-	-	✓	27	46	246	-	-	-	-	-
26	CO	22	W	12	H	279	L	✓	✓	-	-	-	24	48	338	-	-	-	-	-
27	CO	31	W	18	H	491	BI	-	-	-	-	✓	27	48	265	✓	-	-	-	-
28	CO	43	M	14	H	231	C	-	-	✓	-	-	25	47	258	✓	-	-	-	-
29	CO	52	W	10	I	224	L	✓	✓	-	-	-	25	50	134	✓	✓	-	-	-
30	CO	59	M	14	H	228	R	-	-	-	-	✓	26	50	257	✓	-	-	-	-
31	CO	65	M	6	I	187	R	-	-	-	-	✓	28	50	248	✓	✓	-	-	-
32	CO	58	W	9	I	229	BR	-	-	-	✓	-	27	48	239	✓	-	✓	-	-
33	CO	44	W	8	I	188	L	-	✓	-	-	-	24	46	168	✓	✓	-	-	-
34	CO	73	M	18	H	467	R	-	-	-	-	✓	30	50	123	✓	-	-	-	-
35	CO	52	W	12	H	744	BI	-	✓	-	-	-	29	49	206	-	-	-	-	-
36	CO	56	M	12	H	359	R	-	-	-	-	✓	27	48	177	✓	-	✓	✓	-
37	CO	34	M	8	H	1151	R	-	-	-	-	✓	28	47	208	✓	-	-	-	-
38	CO	57	W	17	H	224	BI	-	✓	-	-	-	25	50	393	✓	✓	-	-	-
39	CO	75	W	12	H	253	BI	-	✓	-	-	-	27	46	190	✓	-	-	-	-
40	CO	66	M	15	I	569	L	✓	✓	-	-	-	28	46	121	-	✓	-	-	-
41	CO	19	W	10	H	589	R	-	-	-	-	✓	25	50	191	-	✓	-	-	✓
42	CO	71	M	4	H	366	R	-	✓	-	-	-	27	50	173	✓	-	-	-	-
43	CO	38	M	12	H	320	BI	-	✓	-	-	-	24	48	172	✓	✓	-	✓	✓

Age and time since injury are expressed in days. Education is expressed in years. CO: competitive. NC: non-competitive. M: man. W: woman. I: ischemic stroke. H: hemorrhagic stroke. R: right. L: left. BI: bilateral. C: cerebellum. BR: brainstem. ✓: present. -: absent.

2 Individual intervention-related progress on the D2 Test of Attention

Supplementary Figure 2. The figure depicts the individual progress on the total score of the D2 Test of Attention of all the participants, highlighting the location of their brain lesion.

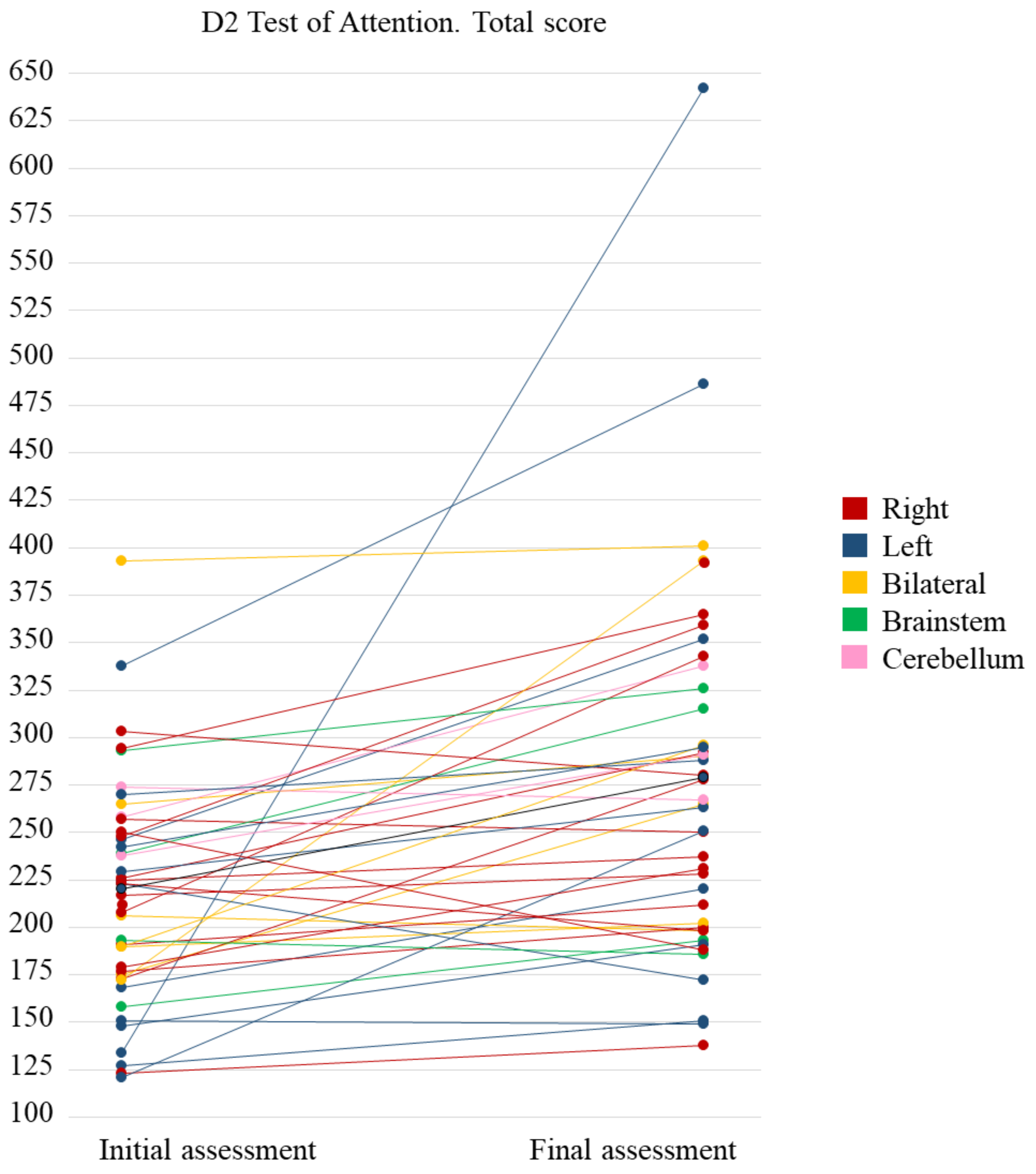

Supplement: Supplementary file 1 [file Data_Sheet_1.PDF]
